# Beta-Blocker Utilization in Intracranial Arteriovenous Malformations: A Narrative Review of Current Evidence, Mechanistic Rationale, and Potential Adjunctive Therapeutic Applications

**DOI:** 10.3390/brainsci16060626

**Published:** 2026-06-11

**Authors:** Thamer Alsharif, Badr Hafiz, Alaa Turkistani, Ziad Alzahrani, Faisal Sukkar, Fahad Okal, Afnan Alkhotani, Mohammed Aref, Mohammed Binmahfoodh, Saleh Baeesa

**Affiliations:** 1 Neurosurgery Department, King Abdulaziz Specialist Hospital, Taif 26521, Saudi Arabia; thameralsharif@outlook.com; 2 Department of Neurosciences, King Faisal Specialist Hospital and Research Centre, Jeddah 21499, Saudi Arabia; neurosurg.badr@gmail.com (B.H.); alaanabil.t@gmail.com (A.T.); ziad.e.sh.alzahrani@gmail.com (Z.A.); aalkhotani@kfshrc.edu.sa (A.A.); maref@kfshrc.edu.sa (M.A.); binmahfoodh@kfshrc.edu.sa (M.B.); 3 Department of Neurosurgery, Dr. Sulaiman Al Habib Medical Group Holding Company, Jeddah 23618, Saudi Arabia; sukkarfaisal@gmail.com; 4 Department of Neurosurgery, King Abdulaziz Medical City, National Guard Health Affairs, Jeddah 22384, Saudi Arabia; dr.3okal@gmail.com; 5 Faculty of Medicine, Alfaisal University, Riyadh 11533, Saudi Arabia

**Keywords:** cerebral arteriovenous malformations, beta-blockers, propranolol, hemorrhage risk, hemodynamic modulation, vascular malformations, cavernous malformation

## Abstract

**Highlights:**

**What are the main findings?**

- Beta-blockers demonstrate potential antiangiogenic and hemodynamic effects that may theoretically influence intracranial arteriovenous malformation biology through modulation of VEGF, HIF-1α, MMP-9, and related signaling pathways.
- Current clinical evidence remains limited to retrospective studies, perioperative reports, and indirect evidence from cerebral cavernous malformations, with no definitive proof of disease-modifying benefit in intracranial AVMs.

**What are the implications of the main findings?**

- Beta-blockers may serve as useful adjuncts for perioperative hemodynamic stabilization in selected intracranial AVM patients.
- Prospective multicenter studies and randomized clinical trials are needed to determine whether beta-blockade can reduce hemorrhage risk or alter AVM progression.

**Abstract:**

**Background/Objective:** Intracranial arteriovenous malformations (AVMs) are high-flow cerebrovascular lesions associated with a significant risk of intracranial hemorrhage, neurological morbidity, and mortality. Current management strategies, including microsurgical resection, endovascular embolization, stereotactic radiosurgery, and conservative observation, remain limited by procedural risk and uncertain long-term outcomes. Beta-blockers, particularly propranolol, have recently attracted interest as potential adjunctive therapies because of their vasoconstrictive, antiangiogenic, and vascular remodeling properties. This review evaluates the mechanistic rationale and current evidence regarding beta-blocker use in intracranial AVMs. **Methods:** A comprehensive literature review was conducted using PubMed, Scopus, and Google Scholar databases through January 2026 using combinations of the terms “arteriovenous malformation,” “AVM,” “beta-blocker,” “propranolol,” “angiogenesis,” “hemorrhage,” and “cerebral cavernous malformation.” Eligible studies included experimental investigations, translational studies, observational cohorts, case reports, clinical trials, systematic reviews, and meta-analyses evaluating beta-blocker use in intracranial AVMs or related vascular malformations. Studies unrelated to cerebrovascular lesions, duplicate reports, and non-English publications were excluded. Given the heterogeneity and limited volume of available AVM-specific literature, findings were synthesized narratively rather than through formal systematic review methodology. **Discussion:** Preclinical studies suggest that beta-blockers modulate molecular pathways implicated in AVM pathophysiology, including VEGF, HIF-1α, SDF1α/CXCR4, MMP-9, and Notch-associated signaling. These mechanisms may reduce abnormal angiogenesis, endothelial instability, and pathological vascular remodeling. Clinical evidence, however, remains limited to retrospective studies, perioperative reports, and indirect evidence from cerebral cavernous malformations. Observational studies have reported associations between beta-blocker exposure and certain favorable AVM characteristics, including lower rates of hemorrhagic presentation and less complex angioarchitecture. However, these findings are highly susceptible to confounding, reverse causation, and selection bias and should not be interpreted as evidence of disease modification. **Conclusions:** Beta-blockers cannot currently be recommended as definitive therapy for intracranial AVMs. Their established role remains perioperative hemodynamic control, while potential disease-modifying effects require validation through prospective studies and randomized clinical trials.

## 1. Introduction

Intracranial arteriovenous malformations (AVMs) are congenital vascular lesions comprising a tangled network of dysplastic vessels that shunt blood directly from arterial to venous circulation, bypassing the normal capillary bed. This high-flow, low-resistance circuitry deprives the surrounding brain parenchyma of normal perfusion and creates a lifelong predisposition to intracranial hemorrhage. Population-based studies estimate the detection rate of symptomatic AVMs at approximately 0.89 to 1.3 per 100,000 person-years, with a point prevalence in adults of roughly 10 to 18 per 100,000 [1,2]. Although AVMs account for only 1.4% to 2% of all strokes, they are a leading cause of nontraumatic intracerebral hemorrhage in young adults, carrying a substantial burden of long-term disability and mortality [1,2].

The clinical presentation of intracranial AVMs is heterogeneous. Approximately 48% of patients initially present with hemorrhage, while others may experience seizures, chronic headaches, or progressive focal neurological deficits attributable to vascular steal phenomena [1]. A substantial proportion of these AVMs are discovered incidentally on neuroimaging performed for unrelated indications. The annual risk of hemorrhage from an unruptured AVM is generally estimated to be 1–3%, although this risk is modified by specific angioarchitectural features, including deep venous drainage, associated intranidal or flow-related aneurysms, deep or infratentorial location, and prior hemorrhage [2].

The contemporary management of intracranial AVMs is multimodal, encompassing microsurgical resection, endovascular embolization, stereotactic radiosurgery, and conservative observation with medical management of associated symptoms. Treatment selection is guided by lesion size, location, venous drainage pattern, and eloquence of adjacent brain parenchyma, most commonly codified through the Spetzler–Martin grading scale, a five-tier system introduced in 1986 that remains the standard for risk stratification [3]. Despite technical advances, each therapeutic modality carries inherent risks. Microsurgical resection, while offering definitive obliteration, carries the risk of postoperative neurological deficits and the phenomenon of normal perfusion pressure breakthrough, resulting in cerebral hyperemia and edema. Radiosurgery entails a latency period of two–three years before complete obliteration, during which the risk of hemorrhage persists. Embolization is often incomplete as a standalone treatment and carries the risk of ischemic stroke and inadvertent occlusion of normal vessels [3].

The management of unruptured intracranial AVMs remains a controversial topic. The ARUBA trial demonstrated lower rates of death or symptomatic stroke with medical management alone compared with interventional therapy during short-term follow-up, although concerns regarding patient selection, treatment heterogeneity, and limited follow-up have been raised [4]. Nevertheless, these findings highlighted the need for less invasive adjunctive therapies that may favorably influence AVM biology or hemorrhage risk without exposing patients to procedural morbidity [4].

More recent comparative-effectiveness analyses have highlighted the complexity of treatment decision-making in contemporary AVM practice. Propensity-matched observational studies have demonstrated that outcomes vary substantially according to AVM grade, rupture status, treatment modality, and patient selection, reinforcing the need for individualized management strategies and continued exploration of adjunctive therapies that may complement existing interventions [5,6].

Interest in beta-blockers originated from the observation that propranolol accelerates the involution of infantile hemangiomas [7]. However, infantile hemangiomas are biologically distinct from AVMs. Hemangiomas are proliferative vascular tumors characterized by endothelial hyperplasia, whereas AVMs are congenital high-flow arteriovenous shunts with fundamentally different hemodynamic and structural properties. Therefore, the therapeutic responses observed in hemangiomas cannot be directly extrapolated to AVMs. Nevertheless, propranolol’s antiangiogenic, vasoconstrictive, and vascular remodeling effects have prompted investigations into whether selected molecular pathways may overlap between these lesions [8]. This narrative review examines the current and potential role of beta-blockers in the management of intracranial AVMs, with a particular focus on hemodynamic control, hemorrhage risk modulation, lesion characteristics, and perioperative adjunctive use. Importantly, the current evidence supporting beta-blocker therapy for intracranial AVMs remains preliminary. Most available data are derived from retrospective observational studies, isolated case reports, experimental models, and extrapolation from other vascular anomalies, such as infantile hemangiomas and cerebral cavernous malformations. Consequently, no definitive conclusions regarding the efficacy or disease-modifying benefits of AVMs can currently be established [8,9,10,11,12,13].

A comprehensive literature review was conducted using PubMed, Scopus, and Google Scholar databases through January 2026 using combinations of the terms “arteriovenous malformation,” “AVM,” “beta-blocker,” “propranolol,” “angiogenesis,” “hemorrhage,” and “cerebral cavernous malformation.” Experimental, translational, and clinical studies relevant to beta-blocker use in intracranial AVMs and related vascular malformations were reviewed and narratively synthesized.

Importantly, the current evidence supporting beta-blocker therapy for intracranial AVMs remains preliminary. Most available data are derived from retrospective observational studies, isolated case reports, experimental models, and extrapolation from related vascular anomalies, including infantile hemangiomas and cerebral cavernous malformations. Consequently, no definitive conclusions regarding efficacy or disease-modifying benefits in AVMs can currently be established.

## 2. Pathophysiology of Intracranial Arteriovenous Malformations

The pathogenesis of intracranial AVMs is complex and multifactorial, involving an interplay between genetic predisposition, aberrant vasculogenesis, and sustained hemodynamic stress. While most AVMs occur sporadically, a subset arises in the context of hereditary syndromes, such as hereditary hemorrhagic telangiectasia, which is associated with mutations in the endoglin and activin receptor-like kinase 1 genes, both of which encode proteins integral to transforming growth factor beta signaling in endothelial cells. These genetic insights have illuminated the broader molecular circuitry that may also be relevant to sporadic AVM formation [14].

At the hemodynamic level, AVMs are characterized by a high-flow, low-resistance shunt that diverts arterial blood directly into the venous circulation, thereby reducing perfusion pressure in the adjacent normal brain parenchyma and generating aberrant wall shear stress on the endothelial lining of the nidal vessels. Pathological shear stress is increasingly recognized as a critical mechanotransduction stimulus that perpetuates vascular remodeling and lesion progression. A landmark study by Rossitti demonstrated that cerebral arteries supplying AVMs undergo progressive dilation but maintain a constant level of wall shear stress, implying that flow-induced shear stress is the driving force for vascular network remodeling in the presence of an AVM [15]. In an experimental rat model, high blood flow conditions in the feeding artery of brain AVMs were shown to induce hemodynamic injury and vascular remodeling, with RNA sequencing revealing key genes and pathways involved in immune and inflammatory infiltration as well as vascular smooth muscle cell phenotype transformation [16]. Elevated shear stress activates endothelial cells to upregulate a host of proangiogenic factors, thereby creating a positive feedback loop in which flow-induced signaling drives further vascular recruitment and nidal expansion.

VEGF, a potent angiogenic cytokine that promotes endothelial cell proliferation, migration, and tube formation, is a central molecular mediator of AVM pathogenesis. Human AVM tissue consistently demonstrates elevated VEGF expression compared to normal cerebral vasculature [17]. VEGF signaling is tightly linked to the HIF 1α pathway, which is activated under conditions of tissue hypoxia and shear stress and serves as a master transcriptional regulator of angiogenic and vasculogenic gene programs. Immunohistochemical analysis of surgical specimens from 26 patients undergoing AVM resection demonstrated that HIF 1α is expressed in human cerebral AVMs and that its expression is significantly associated with VEGF and VEGF receptor expression, suggesting a role for HIF 1α in maintaining angiogenesis and vascular remodeling in these lesions [18]. Experimental models have further shown that cerebral venous hypertension, a condition intimately related to AVM hemodynamics, results in a proangiogenic state in the brain through upregulation of HIF 1α and its downstream targets VEGF and SDF 1α, accompanied by increased leukocyte infiltration and MMP 9 activity [19].

Downstream effectors of the VEGF/HIF 1α axis include matrix metalloproteinases (MMPs), particularly MMP 9, which facilitate degradation of the extracellular matrix and contribute to the structural instability of AVM vessel walls [20]. An additional molecular pathway implicated in AVM pathogenesis is the SDF1α/CXCR4 signaling axis. SDF1α, also known as CXCL12, is a chemokine that recruits circulating endothelial progenitor and inflammatory cells to sites of vascular injury or remodeling. In AVM tissue, SDF1α expression is upregulated and promotes vasculogenesis through CXCR4 dependent pathways that intersect with HIF 1α signaling [8]. This axis represents a putative therapeutic target that may be amenable to pharmacological modulation in the future.

Endothelial dysfunction and endothelial-to-mesenchymal transition (EndMT) also contribute to the instability of AVMs. These processes disrupt endothelial integrity, promote abnormal vascular remodeling, and may increase susceptibility to hemorrhage [14,21]. Dysregulated Notch signaling has also been implicated in the pathogenesis of AVMs. Experimental studies suggest that beta-blockers, such as pronethalol, may suppress Notch-associated pathways involved in endothelial instability and vascular remodeling [22,23].

Collectively, these interconnected molecular pathways, including VEGF/HIF-1α, SDF1α/CXCR4, MMP-9, Notch/RBPJκ/Sox2, and EndMT-related signaling, provide a mechanistic framework through which beta-blockers may theoretically influence biological processes implicated in AVM pathophysiology. However, much of the supporting evidence remains preclinical, and the clinical relevance of these mechanisms has yet to be established.

It should be noted that much of the mechanistic evidence supporting beta-blocker therapy originates from experimental systems, endothelial cell models, infantile hemangioma studies, and related vascular malformations rather than direct investigations of human intracranial AVMs. Consequently, although these findings provide biological plausibility, direct translational evidence supporting disease modification in AVMs remains limited.

## 3. Pharmacologic and Mechanistic Basis of Beta-Blocker Therapy in Intracranial AVMs

Beta-adrenergic receptor antagonists are a pharmacologically diverse class of agents that inhibit the actions of endogenous catecholamines at beta-adrenergic receptors, which are ubiquitously expressed on endothelial cells, vascular smooth muscle cells, and cardiac myocytes. Three distinct beta receptor subtypes exist: beta 1 receptors, predominantly localized in cardiac tissue and juxtaglomerular cells; beta 2 receptors, expressed on vascular smooth muscle and bronchial smooth muscle; and beta 3 receptors, found in adipose tissue and the myocardium. Beta-blockers are classified according to their receptor selectivity (beta 1 selective agents such as atenolol and metoprolol versus nonselective agents such as propranolol and nadolol) and the presence of ancillary properties, including intrinsic sympathomimetic activity, lipophilicity, and combined alpha-adrenergic blockade (for example, labetalol and carvedilol) [24,25,26].

Importantly, the available evidence is not evenly distributed across all beta-blocker subclasses. Most mechanistic and clinical studies have investigated propranolol, a nonselective lipophilic beta-blocker capable of crossing the blood–brain barrier. Comparatively little evidence exists for selective β1-blockers such as metoprolol or atenolol, and essentially no AVM-specific data are available for newer vasodilatory beta-blockers such as nebivolol. Therefore, biological effects observed with propranolol should not automatically be assumed to represent a class effect shared by all beta-blockers.

The therapeutic effects of beta-blockers on vascular anomalies are believed to be mediated through three temporally distinct mechanisms: early vasoconstriction, intermediate inhibition of angiogenesis, and late induction of apoptosis. The vasoconstrictive effect is largely attributable to the antagonism of beta 2 receptor-mediated vasodilation, resulting in unopposed alpha-adrenergic tone and consequent reduction in vessel caliber and blood flow. In high-flow AVMs, such a reduction in flow may attenuate the shear stress stimulus that drives ongoing vascular remodeling [15,16,27].

The antiangiogenic effect of beta-blockers involves the downregulation of key proangiogenic growth factors and signaling intermediates. In a landmark in vitro study, propranolol was shown to dose-dependently inhibit growth factor-induced proliferation of human umbilical vein endothelial cells, suppress chemotactic motility, and inhibit differentiation into capillary-like tubular structures in Matrigel, while also reducing VEGF-induced tyrosine phosphorylation of VEGF receptor 2 and downstream activation of extracellular signal-regulated kinase [28]. In ex vivo experiments using cerebral AVM tissue cultured in the presence of 100 μM propranolol, significant suppression of VEGF and HIF 1α expression at the messenger RNA level was observed [18,27]. Complementary evidence from embryonic stem cell models has demonstrated that beta-blockers, including propranolol, atenolol, and the beta 2 selective antagonist ICI 118,551, dose-dependently downregulate the formation of capillary structures and decrease the expression of VEGF, HIF 1α, and VEGF receptor 2, with the effect mediated, at least in part, through interference with nitric oxide generation [25].

The SDF1α/CXCR4 pathway is another molecular target of beta-blocker action. Propranolol inhibits SDF1α induced vasculogenesis by suppressing CXCR4 expression, an effect that appears to be mediated, at least in part, through the HIF 1α pathway [8]. By interfering with SDF1α/CXCR4 signaling, beta-blockers may attenuate the recruitment of inflammatory cells and circulating endothelial progenitor cells to the AVM nidus, thereby limiting lesion progression [19].

At the level of extracellular matrix remodeling, propranolol has been shown to significantly reduce MMP 9 secretion from human brain microvascular endothelial cells, with this effect correlated to a decrease in MMP 9 gene expression, partly explained by reduced nucleocytoplasmic export of the mRNA stabilizing factor HuR [26]. Notably, MMP 2 secretion was unaffected, indicating a selective effect on MMP 9, the gelatinase most strongly implicated in AVM vessel wall instability [20]. In clinical studies of infantile hemangioma, serum levels of VEGF, basic fibroblast growth factor, and MMP 9 declined significantly after initiation of propranolol therapy [28]. While these molecular changes were documented in a vascular tumor model distinct from AVMs, they provide proof of principle that beta-blockade can modulate pathways that are also active in vascular malformations.

At the transcriptional level, beta-blockers appear to modulate the Notch signaling cascade. The beta antagonist pronethalol has been shown to reduce RBPJκ expression in cerebral AVM models, which in turn prevents the induction of Sox2, a transcription factor that promotes EndMT and vascular destabilization [23]. This effect is independent of beta-adrenergic receptor occupancy, suggesting that beta-blockers may possess receptor-independent actions that contribute to their therapeutic profile in vascular malformations. Furthermore, recent evidence has demonstrated that propranolol can directly target the transcription factor SOX18, which regulates blood vessel formation, and that this effect is independent of its beta-adrenergic receptor blocking activity [27]. The SOX18 pathway has been implicated in multiple vascular anomalies and may represent an additional off-target mechanism through which propranolol influences vascular malformation biology [27].

**Figure 1:**
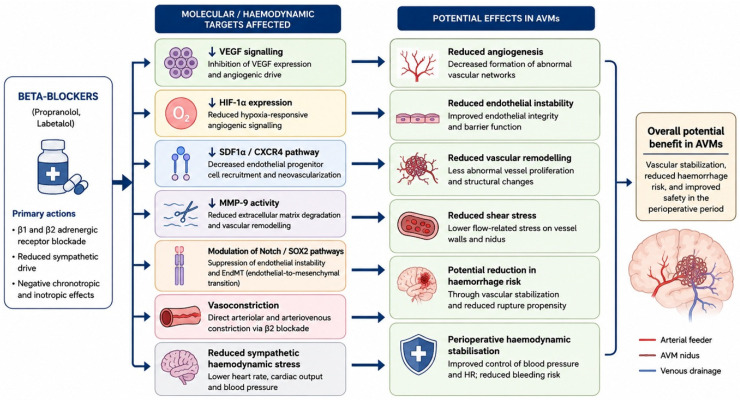
Molecular and hemodynamic mechanisms of beta-blockers in intracranial arteriovenous malformations (AVMs). Beta-blockers may exert vasoconstrictive, antiangiogenic, and hemodynamic effects by modulating VEGF, HIF-1α, SDF1α/CXCR4, MMP-9, and Notch-associated signaling pathways. These mechanisms may theoretically contribute to reduced endothelial instability, decreased vascular remodeling, attenuation of shear stress, perioperative hemodynamic stabilization, and possibly reduced hemorrhage risk, although these effects remain unproven clinically. AVM, arteriovenous malformation; VEGF, vascular endothelial growth factor; HIF-1α, hypoxia-inducible factor-1 alpha; SDF1α, stromal cell-derived factor-1 alpha; MMP-9, matrix metalloproteinase-9. These mechanisms may theoretically contribute to reduced endothelial instability, decreased vascular remodeling, attenuation of shear stress, perioperative hemodynamic stabilization, and possibly reduced hemorrhage risk, although these effects remain unproven clinically. The supporting evidence for these mechanisms originates from a combination of AVM tissue studies, endothelial cell models, infantile hemangioma research, and cerebral cavernous malformation studies, with varying degrees of direct relevance to intracranial AVMs.

The hemodynamic effects of beta-blockers are of particular relevance to the perioperative management of intracranial AVMs. Beta-blockers lower systemic arterial pressure and attenuate the pulsatile stress transmitted to the fragile nidal vasculature by reducing the heart rate, myocardial contractility, and cardiac output. Agents with combined alpha- and beta-adrenergic antagonism, such as labetalol, are especially suited to this context because the alpha blockade component induces peripheral vasodilation, while the beta blockade component blunts reflex tachycardia, yielding controlled hypotension. Critically, studies in healthy humans have demonstrated that labetalol infusion does not influence global or regional cerebral blood flow or cerebral oxygen metabolism, and that cerebral autoregulation is preserved [29]. This profile of preserved cerebral hemodynamics in the setting of systemic blood pressure reduction makes labetalol a particularly attractive agent for patients undergoing neurosurgical procedures for AVMs [26]. The key preclinical findings that underpin the mechanistic rationale for beta-blocker use in AVMs are summarized in Table 1. The molecular and hemodynamic mechanisms through which beta-blockers may influence AVM biology are summarized in Figure 1.

Importantly, the mechanistic evidence supporting beta-blocker therapy is derived from several distinct disease models and should be interpreted accordingly. Suppression of VEGF and HIF-1α expression has been demonstrated in ex vivo AVM tissue studies [27]. In contrast, inhibition of endothelial proliferation, angiogenesis, tubulogenesis, and MMP-9 secretion has been demonstrated primarily in endothelial cell systems and infantile hemangioma models. Similarly, SOX18-mediated effects have been described predominantly in infantile hemangioma research [27]. Evidence from CCM studies contributes primarily to the clinical discussion regarding hemorrhagic outcomes rather than mechanistic understanding of AVM biology. Consequently, the strength and direct applicability of the available evidence vary considerably across the proposed mechanisms, and many observations remain indirect with respect to human intracranial AVMs.

## 4. Current Clinical Evidence

The available clinical evidence supporting beta-blocker therapy in intracranial AVMs remains limited. Consequently, some of the discussion in this review incorporates evidence derived from cerebral cavernous malformations (CCMs). However, CCMs and AVMs are biologically distinct cerebrovascular lesions that differ substantially in vascular architecture, hemodynamics, molecular pathogenesis, and natural history. Therefore, findings derived from CCM studies should be considered indirect and hypothesis-generating rather than directly applicable to AVMs.

### 4.1. Preoperative Use

The preoperative administration of beta-blockers in patients with intracranial AVMs has been investigated primarily in the context of blood pressure control and mitigation of perioperative hemodynamic fluctuations. Contemporary perioperative protocols for AVM resection frequently recommend a vasodilating beta-blocker or calcium channel blocker for one to two weeks postoperatively to maintain systolic blood pressure within a prespecified range and prevent sudden hypertensive surges that may precipitate cerebral hyperperfusion or hemorrhage. For patients with large, high-flow AVMs, more stringent blood pressure control is advocated, with systolic blood pressure targets of 90–110 mmHg in the immediate postoperative period, gradually liberalized over several days [31,32].

The physiological rationale for preoperative beta blockade rests on the premise that chronic attenuation of sympathetic tone may condition the cerebral vasculature to better tolerate the abrupt hemodynamic changes that accompany AVM resection. When the low-resistance AVM nidus is removed, arterial blood that had previously been diverted through the shunt is redirected into the adjacent normal capillary beds, which may have lost their autoregulatory capacity due to chronic hypoperfusion [14]. The resulting hyperperfusion, termed normal perfusion pressure breakthrough, can manifest as cerebral edema, hemorrhage, and neurological deterioration. Beta-blockers may theoretically reduce the severity of this phenomenon by blunting both systemic pressure and the myocardial hyperdynamic response. Additionally, the alpha- and beta-adrenergic antagonists labetalol and esmolol have been identified as ideal hypotensive agents for AVM surgery because they produce predictable, titratable reductions in systemic pressure with minimal effects on cerebral circulation [29,33].

Direct clinical evidence supporting preoperative beta-blocker initiation specifically for AVM surgery is limited to anecdotal reports. One case report described the successful intraoperative use of intravenous propranolol (0.2 mg) to control acute hyperemia and brain swelling that occurred immediately after total AVM resection and was refractory to conventional antihypertensive agents, including nicardipine, diltiazem, and sodium nitroprusside [34]. The propranolol bolus produced a prompt reduction in blood pressure and resolution of brain swelling, with jugular bulb oxygen saturation monitoring providing objective evidence of improved cerebral hemodynamics. While this case is instructive, it does not establish efficacy, and no randomized controlled trial has evaluated preoperative beta-blocker initiation for intracranial AVM surgery [34,35].

### 4.2. Hemodynamic Modulation

The capacity of beta-blockers to modulate cerebral and systemic hemodynamics in the context of intracranial AVMs is central to their proposed therapeutic role. Beta-blockers decrease the pressure gradient across the AVM nidus by reducing the cardiac output, heart rate, and systemic arterial pressure, which in turn is expected to reduce the flow through the shunt and diminish the shear stress acting on dysplastic vessel walls. Given the established role of shear stress in driving vascular remodeling of AVM feeding arteries, sustained hemodynamic modulation could, in principle, slow the cycle of vascular remodeling and nidal expansion that characterizes progressive AVMs [15,16]. However, direct quantitative evidence of flow reduction in human AVMs following beta-blocker administration is currently lacking.

The most comprehensive clinical data to date come from a retrospective analysis of 483 patients from two vascular centers over a 20 year period, of whom 73 (15%) were receiving beta-blocker therapy [8]. Patients on beta-blockers had a significantly higher likelihood of presenting with a lower Spetzler–Martin grade (grade III or lower; *p* < 0.0001; odds ratio 6.5) and a lower prevalence of associated aneurysms (*p* < 0.0001; odds ratio 3.6) than those not receiving beta-blockers. These observations suggest an association between beta-blocker use and a less complex AVM angioarchitecture. However, reverse causation and confounding by indication remain important alternative explanations. Patients receiving beta-blockers often differ substantially from untreated patients with respect to age, cardiovascular comorbidities, healthcare utilization, and concomitant medications. Consequently, these findings should not be interpreted as evidence that beta-blockers directly modify AVM morphology or disease progression. It must be emphasized that the cross-sectional design cannot distinguish between a true therapeutic effect on lesion progression and a selection phenomenon, whereby patients with less severe vascular pathology are more likely to be prescribed beta-blockers for unrelated cardiovascular indications [29]. Importantly, this study is currently available only in conference abstract form, precluding detailed assessment of patient selection, variable definitions, statistical modelling, and residual confounding. Consequently, its findings should be regarded as hypothesis-generating rather than confirmatory evidence.

The hemodynamic effects of beta-blockers may also extend to the venous compartment of the AVM. By reducing arterial inflow, beta-blockers secondarily decrease venous outflow pressure and flow velocity, potentially mitigating the risk of venous outflow obstruction or thrombosis, which has been implicated as a precipitant of AVM hemorrhage [14]. Quantitative hemodynamic studies using catheter angiography or phase-contrast magnetic resonance imaging to directly measure changes in nidal flow before and after beta-blocker initiation have not been performed, and the magnitude of flow reduction achievable with clinical dosing remains undefined.

### 4.3. Hemorrhage Related Considerations

Reducing the risk of hemorrhagic stroke is the principal objective in AVMs management, and the possibility that beta-blockers might contribute to this goal represents the most clinically significant aspect of their emerging therapeutic profile. The pathophysiological basis for this potential protective effect is plausible: beta-blockers lower systemic arterial pressure and attenuate the hemodynamic stress imposed on structurally fragile nidal vessels that lack the smooth muscle investment and elastic lamina integrity of normal cerebral arteries. Additionally, beta blockade mediated suppression of MMP 9 may reduce vessel wall degradation, and downregulation of VEGF and HIF 1α may stabilise the vascular architecture [8,20,26]. It is important to state clearly that direct evidence of hemorrhage prevention by beta blockers in human AVMs is not available, and the notion remains a hypothesis requiring rigorous prospective evaluation.

#### 4.3.1. Evidence in True Intracranial AVMs

In the retrospective cohort described above, 48% of all AVMs presented with hemorrhage [8]. On univariate analysis, beta-blocker use was associated with a significantly lower risk of hemorrhagic presentation (*p* < 0.0001; odds ratio 13). This association persisted in the multivariate analysis adjusted for Spetzler–Martin grade, age ≤ 50 years, and presence of associated aneurysm, with beta-blocker use independently associated with reduced hemorrhage risk (*p* < 0.01; odds ratio 0.2). Although these findings are intriguing, they warrant cautious interpretation. Notably, the apparent association changed substantially between the univariate and multivariable analyses, with the reported odds ratio shifting from 13 in unadjusted analyses to 0.2 after adjustment for selected covariates. Such a marked change suggests the presence of important confounding factors and highlights the sensitivity of the observed association to statistical adjustment. Furthermore, the study is retrospective and currently available only in abstract form, limiting detailed appraisal of the methodology and robustness of the findings. Accordingly, these observations should be considered exploratory and hypothesis-generating rather than evidence of a causal protective effect.

#### 4.3.2. Indirect Evidence from Cerebral Cavernous Malformations

Cerebral cavernous malformations (CCMs) differ substantially from AVMs in terms of vascular architecture, hemodynamics, molecular pathogenesis, and clinical natural history. Unlike AVMs, which are high-flow arteriovenous shunts characterized by direct arterial-to-venous connections, CCMs consist of low-flow vascular caverns lacking intervening neural tissue. Although both lesions exhibit abnormalities in vascular biology and angiogenic signaling, the overlap is only partial. Consequently, findings from CCM studies should be interpreted as indirect supportive evidence and hypothesis-generating observations rather than direct evidence of efficacy in AVMs. Cerebral cavernous malformations differ substantially from AVMs in terms of vascular architecture, hemodynamics, molecular pathogenesis, and clinical natural history. Although both lesions exhibit abnormalities in vascular biology and angiogenic signaling, the overlap is only partial. Consequently, findings from CCM studies should be interpreted as indirect supportive evidence and hypothesis-generating observations rather than direct evidence of efficacy in AVMs.

Indirect support for a potential protective effect comes from studies of CCMs, which share certain molecular features with AVMs, including dysregulated angiogenesis. In a prospective cohort with up to 15 years of follow up from the Scottish Audit of Intracranial Vascular Malformations, beta blocker use was associated with a significantly lower risk of new intracranial hemorrhage or persistent/progressive focal neurological deficit in patients with CCMs (adjusted hazard ratio 0.09) [9]. The Treat_CCM randomized phase 2 pilot trial evaluated propranolol in 83 patients with familial CCMs and found a lower incidence of symptomatic intracerebral hemorrhage or focal neurological deficit in the propranolol group (1.7 per 100 person years) compared with standard care alone (3.9 per 100 person years; hazard ratio 0.43, 80% confidence interval 0.18 to 0.98) [10]. Although the trial was not designed to be adequately powered for efficacy, the prespecified efficacy signal supports further investigation. A more recent systematic review and meta analysis of five studies encompassing 1553 participants found that beta blocker exposure was associated with significantly lower odds of new onset intracerebral hemorrhage or nonhemorrhagic focal neurological deficit attributable to CCMs (odds ratio 0.52, 95% confidence interval 0.35 to 0.77; *p* = 0.001) [11]. However, an earlier meta-analysis limited to different studies had failed to demonstrate a statistically significant protective effect (overall effect 0.78, 95% confidence interval 0.20 to 3.11; *p* = 0.73) [13]. The applicability of these CCM data to true AVMs is uncertain, given the profound hemodynamic differences between the two lesion types.

### 4.4. Adjunctive Peri-Interventional Role

Beyond any potential chronic effects, beta-blockers may serve as valuable adjunctive agents during and immediately after interventional procedures for intracranial AVMs. The peri-interventional period is characterized by heightened vulnerability to hemodynamic perturbations, and pharmacological stabilization of blood pressure and cardiac output represents a rational strategy to prevent secondary neurological injury.

During microsurgical AVM resection, deliberate controlled hypotension is frequently employed to reduce blood loss, facilitate dissection, and minimize the risk of intraoperative rupture. An ideal hypotensive agent should produce predictable, titratable reductions in systemic pressure without impairing cerebral autoregulation or causing rebound hypertension. Alpha- and beta-adrenergic antagonists, such as labetalol and the short acting beta 1 selective agent esmolol, fulfil these criteria and have been recommended as first-line agents for controlled hypotension during AVM surgery [35]. The preservation of cerebral blood flow and autoregulation documented with labetalol makes it particularly suitable for this neurosurgical context [29].

The anecdotal use of intravenous propranolol to manage refractory postoperative hyperemia, as described in Section 4.1, further illustrates the potential peri-interventional role of beta blockade [34]. Postoperative blood pressure management is a critical determinant of the outcome. Patients with medium to large AVMs or those with small AVMs complicated by intraoperative hemorrhage or technical difficulty are typically maintained on a vasodilating beta-blocker or calcium channel blocker for one to two weeks after surgery, with systolic blood pressure targets below 120 to 130 mmHg. For high-risk patients with large, complex AVMs, more intensive regimens that include sedation and prolonged strict blood pressure control have been described [31].

During endovascular embolization procedures, abrupt changes in flow dynamics after partial nidal occlusion can alter the pressure gradients within the residual AVM, potentially increasing the risk of hemorrhage. Beta-blockers may help stabilize systemic hemodynamics during this period and blunt the sympathetic response to procedural stimulation. The European consensus conference on unruptured brain AVMs has emphasized the importance of a multidisciplinary approach to treatment planning, within which perioperative medical management, including blood pressure control, plays an integral role [36].

A systematic review of beta-blocker therapy in AVMs and related vascular lesions, encompassing 18 studies, concluded that current evidence does not establish beta-blockers as a definitive primary therapy for true AVMs but noted that in selected AVM cases, beta-blockers may provide symptomatic benefit or short-term stabilization [12]. The review emphasized that the evidence base is dominated by infantile hemangioma studies and that true AVM-specific evidence is limited to case reports and small retrospective series. The adjunctive peri-interventional role of beta-blockers, although supported by expert consensus and institutional protocols, has not been subjected to rigorous comparative effectiveness research. The optimal agent, dose, timing of initiation, duration of therapy, and target hemodynamic parameters remain to be defined through prospective investigations. The clinical evidence on beta-blockers- and intracranial AVMs, including supportive data from cerebral cavernous malformation trials, is compiled in Table 2.

## 5. Lesion Characteristics and Potential Treatment Stratification

The observation that beta-blocker use is associated with lower Spetzler–Martin grade lesions and a reduced prevalence of associated aneurysms invites consideration of whether these agents could be integrated into a stratified treatment paradigm [6]. Although the retrospective nature of the data precludes causal inference, the mechanistic properties of beta-blockers offer a biologically coherent explanation for such associations. The Spetzler–Martin grading system incorporates nidal size, venous drainage pattern, and eloquence of the adjacent brain, and has remained the cornerstone of AVM risk stratification since its introduction [3]. A shift toward lower grades implies a smaller nidal diameter, superficial rather than deep venous drainage, or both. Deep venous drainage has consistently been identified as an important predictor of hemorrhagic presentation and adverse natural history in intracranial AVMs, making this component of the Spetzler–Martin grading system particularly relevant when considering potential disease-modifying interventions [3,37].

The antiangiogenic effects of beta-blockers may attenuate the chronic vascular remodeling that drives nidal enlargement. By downregulating VEGF and HIF 1α expression, propranolol interferes with the core signaling axis responsible for endothelial proliferation and new vessel formation [17,18]. Suppression of the SDF1α/CXCR4 pathway may further limit the recruitment of circulating endothelial progenitor cells, which contribute to lesion growth and complexity [8].

To date, no human imaging study has demonstrated AVM regression, reduction in nidus size, or alteration of natural history attributable to beta-blocker therapy. Consequently, any proposed stabilizing effect remains speculative and should be considered hypothesis-generating until validated in prospective studies.

The significantly lower prevalence of associated aneurysms in patients receiving beta blockers (odds ratio 3.6) is of particular interest because feeding artery and intranidal aneurysms are among the strongest independent predictors of hemorrhage [8]. The hemodynamic hypothesis posits that sustained reductions in systemic arterial pressure and nidal flow would diminish the pulsatile wall stress that contributes to aneurysm formation in dysplastic feeding vessels. This concept aligns with the fundamental role of shear stress in arterial remodeling, as demonstrated in AVM feeding vessels [15]. However, it remains entirely possible that patients with less complex vascular anatomy were simply more likely to be prescribed beta-blockers for unrelated cardiovascular indications, such as hypertension or coronary artery disease, creating a spurious association.

Any future stratification framework would require detailed angioarchitectural phenotyping and serial imaging to determine whether beta-blocker exposure genuinely modifies lesion morphology over time. Patients with diffuse, high-flow lesions that carry unacceptable risks for definitive intervention might theoretically derive the greatest benefit from a pharmacological approach aimed at slowing progression, whereas those with small, surgically accessible lesions might not require such an adjunct. These considerations are speculative and must not be interpreted as evidence that beta-blockers reduce lesion size or complexity in human intracranial AVMs.

### Potential Risks and Safety Considerations

Any consideration of beta-blockers as long-term adjunctive therapy for AVMs must be balanced against their known adverse-effect profile. Many AVM patients are young and normotensive, and chronic beta-blockade may expose them to treatment-related morbidity without proven benefit. Common adverse effects include fatigue, exercise intolerance, dizziness, bradycardia, hypotension, sexual dysfunction, and sleep disturbance [24,37]. Depression and mood-related symptoms have also been reported, although causality remains debated [36,37]. Nonselective agents such as propranolol may precipitate bronchospasm in susceptible individuals and should be used cautiously in patients with reactive airway disease [24,37]. Abrupt discontinuation can produce rebound sympathetic activation, resulting in tachycardia and hypertension [37]. These considerations are particularly important when contemplating prolonged therapy for an indication in which efficacy remains unproven. Future clinical trials should incorporate systematic assessment of adverse events, quality-of-life measures, and treatment adherence alongside efficacy outcomes.

## 6. Limitations of Existing Evidence

The evidence supporting the role of beta-blockers in intracranial AVM management is preliminary and subject to several important limitations that preclude definitive clinical recommendations. First and most critically, no randomized controlled trial has been designed to evaluate beta-blocker therapy specifically in the AVM population. All published clinical data are derived from retrospective observational studies, case reports, and extrapolations from biologically distinct entities such as infantile hemangioma and cerebral cavernous malformations [8,9,10,11].

The largest available clinical dataset is the multicenter retrospective cohort described by Kashefiolasl et al., which included 73 patients receiving beta-blockers among 483 participants [8]. This study is available only in abstract form, which limits the critical appraisal of its methodology, statistical modelling, and handling of confounders. The indications for beta-blocker prescription were not systematically captured, and patients may have been receiving these agents for a wide spectrum of cardiovascular conditions, each associated with distinct demographic and comorbidity profiles that could independently influence the natural history of AVM. Concomitant medications with potential vascular effects, such as statins, antiplatelet agents, and anticoagulants, were not considered in the analysis, representing a significant source of residual confounding.

An additional concern is confounding by indication. Patients prescribed beta-blockers often differ systematically from those not receiving these agents. They are typically older, more likely to have hypertension or cardiovascular comorbidities, may receive concomitant medications such as statins or antiplatelet therapy, and frequently undergo more regular medical surveillance. Any of these factors could independently influence AVM detection, clinical presentation, or hemorrhage risk. Consequently, observed associations between beta-blocker exposure and more favorable AVM characteristics may reflect underlying patient differences rather than a true pharmacological effect. Future studies should employ propensity matching, inverse probability weighting, or randomized allocation to address this important source of bias.

Another major limitation is the complete absence of quantitative hemodynamic data. The mechanistic rationale for beta-blocker therapy depends on the assumption that clinically tolerated doses produce a meaningful reduction in flow through the AVM nidus; however, no study has directly measured nidal hemodynamics before and after beta-blocker initiation using catheter angiography, transcranial Doppler, or phase-contrast magnetic resonance imaging. The magnitude of flow reduction required to alter shear stress signaling or confer hemostatic benefits is unknown.

The heterogeneity of beta-blocker agents represents an additional confounder. Preclinical experiments demonstrating suppression of VEGF, SDF1α, and Notch pathway components were predominantly performed with propranolol or pronethalol [23,25], whereas clinical cohorts included patients receiving a variety of beta-blockers with differing receptor selectivity and pharmacokinetic properties. The effects of a non-selective lipophilic agent cannot be assumed to be interchangeable with those of a beta 1 selective hydrophilic compound, particularly with respect to central nervous system penetration and antiangiogenic potency.

Furthermore, most mechanistic and clinical evidence currently pertains to propranolol. Whether selective β1-blockers or agents with additional vasodilatory properties, such as nebivolol, exert comparable antiangiogenic, vascular remodeling, or hemodynamic effects in AVMs remains unknown. The paucity of comparative data prevents extrapolation of propranolol-specific findings to the broader beta-blocker class and represents an important area for future investigation.

Finally, a systematic review by Alzahrani et al., which evaluated beta-blocker use in AVMs and related vascular lesions across 18 studies, concluded that the existing evidence does not establish beta-blockers as a definitive primary therapy for true AVMs and that the literature is heavily weighted toward infantile hemangioma rather than intracranial arteriovenous shunts. Publication bias and the absence of prospectively registered confirmatory studies raise the possibility that the observed associations may be overestimated. Until these evidentiary gaps are addressed, the use of beta-blockers as disease-modifying agents in intracranial AVMs must remain strictly investigational.

## 7. Future Research Directions

A coordinated research program spanning preclinical investigations, prospective observational studies, and ultimately randomized controlled trials is necessary to define the role of beta-blockers in intracranial AVM management. Several priorities have been identified.

First, prospective observational registries with standardized data collection and serial neuroimaging are essential. Such registries should capture detailed information on the specific beta-blocker agent, dose, duration of therapy, and clinical indication, together with comprehensive angioarchitectural characterization and concurrent medication profiles. Outcomes of interest included AVM hemorrhage, lesional growth or regression, change in Spetzler–Martin grade, and development of new associated aneurysms. Propensity score matching or inverse probability of treatment weighting should be employed to address confounding factors.

Second, quantitative hemodynamic investigations are needed to determine whether beta-blockers produce a measurable reduction in nidal flow. Phase-contrast magnetic resonance angiography offers a noninvasive method for quantifying blood flow in major feeding arteries and draining veins before and after beta-blocker initiation. Correlating the magnitude of flow reduction with molecular markers, such as serum VEGF and MMP 9 levels, could establish a pharmacodynamic profile to guide dosing and agent selection in future trials.

Third, preclinical studies using in vivo AVM models should examine the effects of sustained beta-blockade on nidal vessel density, endothelial proliferation, mural cell coverage, and expression of key molecular mediators, including VEGF, MMP 9, SDF1α, and Notch pathway components [17,26]. The identification of a biomarker signature that correlates with the therapeutic response in animal models would facilitate the design of early phase human trials incorporating surrogate endpoints, thereby reducing the need for prolonged follow-up to capture clinical events.

Fourth, a phase II randomized placebo-controlled trial is the logical next step. Such a trial might enroll patients with unruptured Spetzler–Martin grade I to III AVMs who are being managed conservatively and randomize them to a nonselective beta-blocker, such as propranolol, or placebo. The primary outcome could be a composite of hemorrhage, symptomatic growth, or transition to a higher Spetzler–Martin grade over a follow-up period of two–three years. Secondary outcomes would include quantitative hemodynamic parameters, quality of life measures, and adverse events. The Treat_CCM trial, which evaluated propranolol in familial cerebral cavernous malformations and demonstrated a prespecified signal of efficacy, provides a useful template for the feasibility of such a design in cerebrovascular malformations, although AVMs present distinct challenges owing to their low annual hemorrhage rate [10]. A large multicenter collaboration is essential to achieve adequate statistical power, and the ARUBA trial has already established the feasibility of randomized controlled studies in the AVM population [4]. Nevertheless, AVMs present unique challenges for clinical trial design. The relatively low annual hemorrhage rate of many unruptured lesions necessitates prolonged follow-up and large sample sizes to detect clinically meaningful differences in event rates. Consequently, future trials will likely require multicenter international collaboration, incorporation of surrogate imaging or hemodynamic biomarkers, and careful patient selection to remain feasible.

Fifth, comparative effectiveness studies of different beta-blocker subclasses are warranted. Agents with combined alpha and beta adrenergic antagonism, such as labetalol, may offer superior perioperative hemodynamic control due to their preserved cerebral autoregulation profile [29], whereas the nonselective antiangiogenic profile of propranolol may be better suited for long-term lesion modulation. Head-to-head preclinical comparisons followed by pilot clinical evaluations would help delineate the optimal pharmacologic strategy for distinct clinical contexts, as emphasized by the European consensus conference on unruptured brain AVMs, which called for individualized, multidisciplinary treatment planning [36].

## 8. Conclusions

Propranolol and, to a lesser extent, other beta-blockers have emerged as potential adjunctive agents for intracranial AVMs because of their vasoconstrictive, antiangiogenic, and hemodynamic effects. Experimental evidence supports modulation of pathways implicated in angiogenesis, endothelial instability, and vascular remodeling, including VEGF, HIF-1α, MMP-9, SDF1α/CXCR4, and Notch-associated signaling. However, current clinical evidence remains insufficient to establish a disease-modifying role, consisting primarily of retrospective observational studies, case reports, and indirect evidence from cerebral cavernous malformations. Consequently, beta-blockers cannot currently be recommended as definitive therapy for intracranial AVMs outside investigational settings. Future changes in clinical practice would require robust prospective evidence demonstrating reductions in hemorrhage, AVM progression, or lesion complexity, supported by adequately powered randomized controlled trials and objective hemodynamic or imaging biomarkers. Until such evidence becomes available, the principal role of beta-blockers in AVM care remains perioperative hemodynamic management.

## Figures and Tables

**Table 1:** Preclinical studies investigating beta-blocker effects on angiogenesis, vasculogenesis, and vascular remodeling.

Study (Year)	Experimental Model	Beta-Blocker (Concentration/Dose)	Key Findings	Molecular Targets/Pathways Affected
Lamy et al. (2010) [30]	Human umbilical vein endothelial cells (HUVECs)	Propranolol (dose-dependent)	Inhibited proliferation, chemotactic motility, and capillary-like tube formation; reduced VEGF-induced VEGFR-2 phosphorylation and ERK activation	VEGFR-2, ERK
Annabi et al. (2009) [26]	Human brain microvascular endothelial cells (HBMECs)	Propranolol (10–100 µM)	Suppressed tubulogenesis and selectively reduced MMP-9 secretion without affecting MMP-2; decreased MMP-9 mRNA stability via HuR nuclear retention	MMP-9, HuR
Sharifpanah et al. (2014) [25]	Mouse embryonic stem cells	Propranolol, atenolol, ICI 118,551 (β_2_-selective)	Dose-dependent inhibition of capillary structure formation; downregulated VEGF, HIF-1α, and VEGFR-2; effect partly mediated by reduced nitric oxide generation	VEGF, HIF-1α, VEGFR-2, NO
Qiao et al. (2020) [23]	Cerebral AVM model (pronethalol)	Pronethalol	Decreased RBPJκ expression preventing Sox2 induction; effect persisted after beta-adrenergic receptor depletion	Notch/RBPJκ/Sox2 (receptor-independent)
Seebauer et al. (2022) [27]	Infantile hemangioma (mechanism study)	Propranolol	Identified SOX18 as a direct transcriptional target of propranolol, independent of beta-blockade; SOX18 regulates blood vessel formation	SOX18 (receptor-independent)

**Table 2:** Clinical studies assessing beta-blocker therapy in intracranial AVMs and related vascular malformations.

Study (Year)	Design	Population	Beta-Blocker (When Specified)	Key Outcome(s) Related to Beta-Blockers	Main Limitations
Kashefiolasl et al. (2022) [8]	Retrospective cohort (two centers, 20-year period)	483 patients with intracranial AVMs (73 on beta-blockers)	Not specified (various agents)	Beta-blocker use associated with lower Spetzler-Martin grade (OR 6.5, *p* < 0.0001), fewer associated aneurysms (OR 3.6, *p* < 0.0001), and reduced hemorrhagic presentation (multivariate OR 0.2, *p* < 0.01)	Retrospective design, abstract only, indication for beta-blocker unknown, residual confounding
Kimiwada et al. (2003) [34]	Case report	Single patient with intraoperative hyperemia after AVM resection	Propranolol 0.2 mg IV	Prompt reduction of blood pressure and brain swelling refractory to other agents; improved jugular bulb oxygen saturation	N = 1, no comparison group, cannot establish efficacy
Lanfranconi et al. (2023) [10] (Treat_CCM)	Randomised, open-label, phase 2 pilot trial	83 patients with familial cerebral cavernous malformations (CCMs)	Propranolol (up to 160 mg/day)	Lower incidence of symptomatic ICH or focal neurological deficit: 1.7 vs. 3.9 per 100 person-years (HR 0.43, 80% CI 0.18–0.98)	Not an AVM population; underpowered for definitive efficacy; open-label design
Zuurbier et al. (2022) [9]	Prospective population-based cohort	300 patients with CCMs (SAIVM)	Any beta-blocker	Beta-blocker use associated with lower risk of new ICH or persistent/progressive focal deficit (adjusted HR 0.09)	CCMs, not AVMs; observational; small number of events
Wang et al. (2026) [11]	Systematic review and meta-analysis (5 studies)	1553 participants with CCMs	Various beta-blockers	Beta-blocker exposure associated with reduced odds of new ICH or non-hemorrhagic focal deficit (OR 0.52, 95% CI 0.35–0.77, *p* = 0.001)	Includes only CCM studies; heterogeneity across designs; cannot be directly applied to AVMs
Alzahrani et al. (2026) [12]	Systematic review (18 studies)	AVMs and related vascular lesions	Various beta-blockers	Concluded that evidence does not support beta-blockers as primary AVM therapy; some cases showed symptomatic benefit or short-term stabilization	Predominantly infantile hemangioma studies; very limited true AVM data

## Data Availability

No new data were created or analyzed in this study. Data sharing is not applicable to this article.
